# A New Specimen of the Controversial Chasmosaurine *Torosaurus latus* (Dinosauria: Ceratopsidae) from the Upper Cretaceous Hell Creek Formation of Montana

**DOI:** 10.1371/journal.pone.0151453

**Published:** 2016-03-14

**Authors:** Andrew T. McDonald, Carl E. Campbell, Brian Thomas

**Affiliations:** 1 Earth Sciences, Saint Louis Science Center, Saint Louis, Missouri, United States of America; 2 Geology, St. Louis Community College-Meramec, Saint Louis, Missouri, United States of America; University of Naples, ITALY

## Abstract

*Torosaurus latus* is an uncommon and contentious taxon of chasmosaurine ceratopsid known from several upper Maastrichtian units in western North America. We describe a partial parietal of *To*. *latus* from the Hell Creek Formation of Montana. Although the specimen’s ontogenetic maturity means that it cannot inform the ongoing debate over whether *To*. *latus* is the old adult form of the contemporary *Triceratops*, the specimen is one of the best-preserved *To*. *latus* parietals and supplements previous descriptions.

## Introduction

*Torosaurus latus* [1] is a chasmosaurine ceratopsid from the late Maastrichtian of western North America. It is represented by no more than nine specimens: two or possibly three specimens from the Lance Formation of Wyoming (holotype YPM 1830, YPM 1831, and possibly GP 245–4), three from the Hell Creek Formation of Montana (MOR 981, MOR 1122, and MPM VP6841), two from the Hell Creek Formation of South Dakota (ANSP 15192 and SMM P97.6.1), and possibly one from the Frenchman Formation of Saskatchewan (EM P16.1) [2]. Compared to its late Maastrichtian contemporary *Triceratops*, which is known from dozens of skulls [3, 4], *Torosaurus latus* is extremely rare.

Here, we describe a new specimen, ESU 2009–6, of *Torosaurus latus* from the Hell Creek Formation of Montana. The specimen consists of an incomplete parietal and was discovered in 2009 in Garfield County by David Lukens of the Eastern Missouri Society for Paleontology. It was collected from an overbank clay in the middle part of the upper third of the Hell Creek Formation [5]. ESU 2009–6 is currently reposited at Johnston Geology Museum, Emporia State University in Emporia, Kansas, and is on loan for display at the Saint Louis Science Center in Saint Louis, Missouri, USA.

Recently, it has been proposed that *Torosaurus latus* is not a distinct taxon, but rather represents the old adult form of *Triceratops* [4, 6, 7]; however, this hypothesis has been challenged and the validity of the taxon upheld by other authors [8–10]. The purpose of this paper is not to participate in this continuing debate; however, regardless of whether it is a distinct taxon or the fully mature form of *Triceratops*, *Torosaurus latus* represents a rare morphology, and the description of an additional specimen will be highly beneficial. The specimen will be referred to as *Torosaurus latus* in this paper for ease of communication, with the caveat that *To*. *latus* remains a problematic entity.

### Institutional Abbreviations

ANSP, Academy of Natural Sciences, Philadelphia, PA, USA; EM, Eastend Museum, Eastend, Saskatchewan, Canada; ESU, Emporia State University, Emporia, KS, USA; GP, Glenrock Paleontological Museum, Glenrock, WY, USA; MOR, Museum of the Rockies, Bozeman, MT, USA; MPM, Milwaukee Public Museum, Milwaukee, WI, USA; SMM, Science Museum of Minnesota, St. Paul, MN, USA; YPM, Yale Peabody Museum of Natural History, New Haven, CT, USA.

## Description of ESU 2009–6

**Fig 1:**
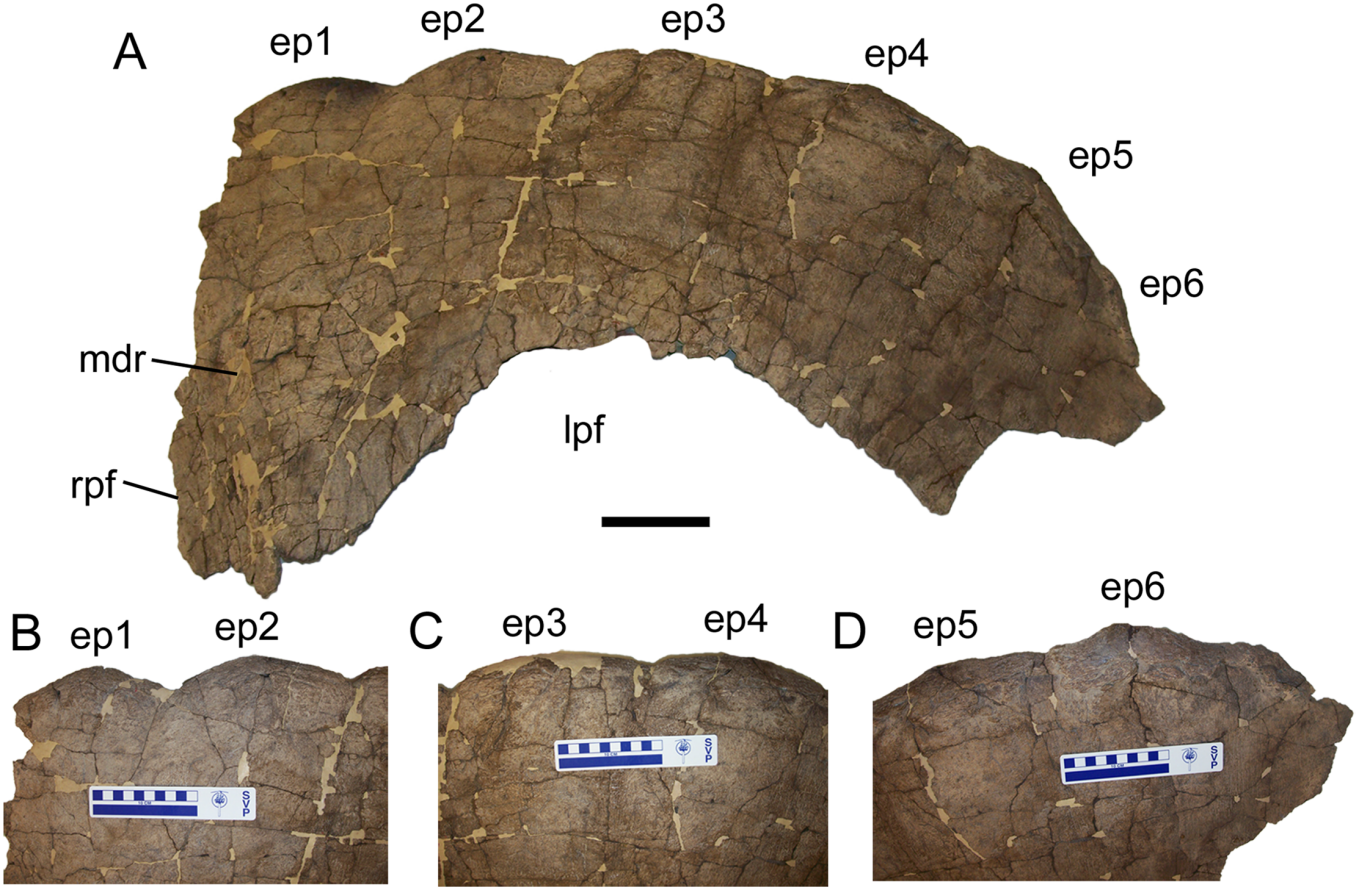
Parietal of *Torosaurus latus*. (A) ESU 2009–6 in dorsal view. (B–D) Epiparietals in dorsal view, including (B) ep1 and ep2, (C) ep3 and ep4, and (D) ep5 and ep6. *Abbreviations*: *ep1*, epiparietal locus 1; *ep2*, epiparietal locus 2; *ep3*, epiparietal locus 3; *ep4*, epiparietal locus 4; *ep5*, epiparietal locus 5; *ep6*, epiparietal locus 6; *lpf*, left parietal fenestra; *mdr*, midline ridge; *rpf*, rim of right parietal fenestra. Scale bars equal 10 cm.

Measurements of ESU 2009–6 are provided in the supplementary information (S1 Table). ESU 2009–6 comprises most of the left side of the parietal, broken immediately medial to the squamosal contact (Fig 1A). The parietal is gently arched dorsally along its transverse axis. Part of the midline parietal bar is preserved; it is mediolaterally broad and bears a subtle midline ridge. A small portion of the medial rim of the right parietal fenestra is preserved (Fig 1A). In contrast, most of the medial and the entire caudal rims of the left parietal fenestra are preserved; this fenestra appears to have been mediolaterally wide, similar to other specimens referred to *Torosaurus latus* (e.g., MOR 981, MOR 1122) [2].

**Fig 2:**
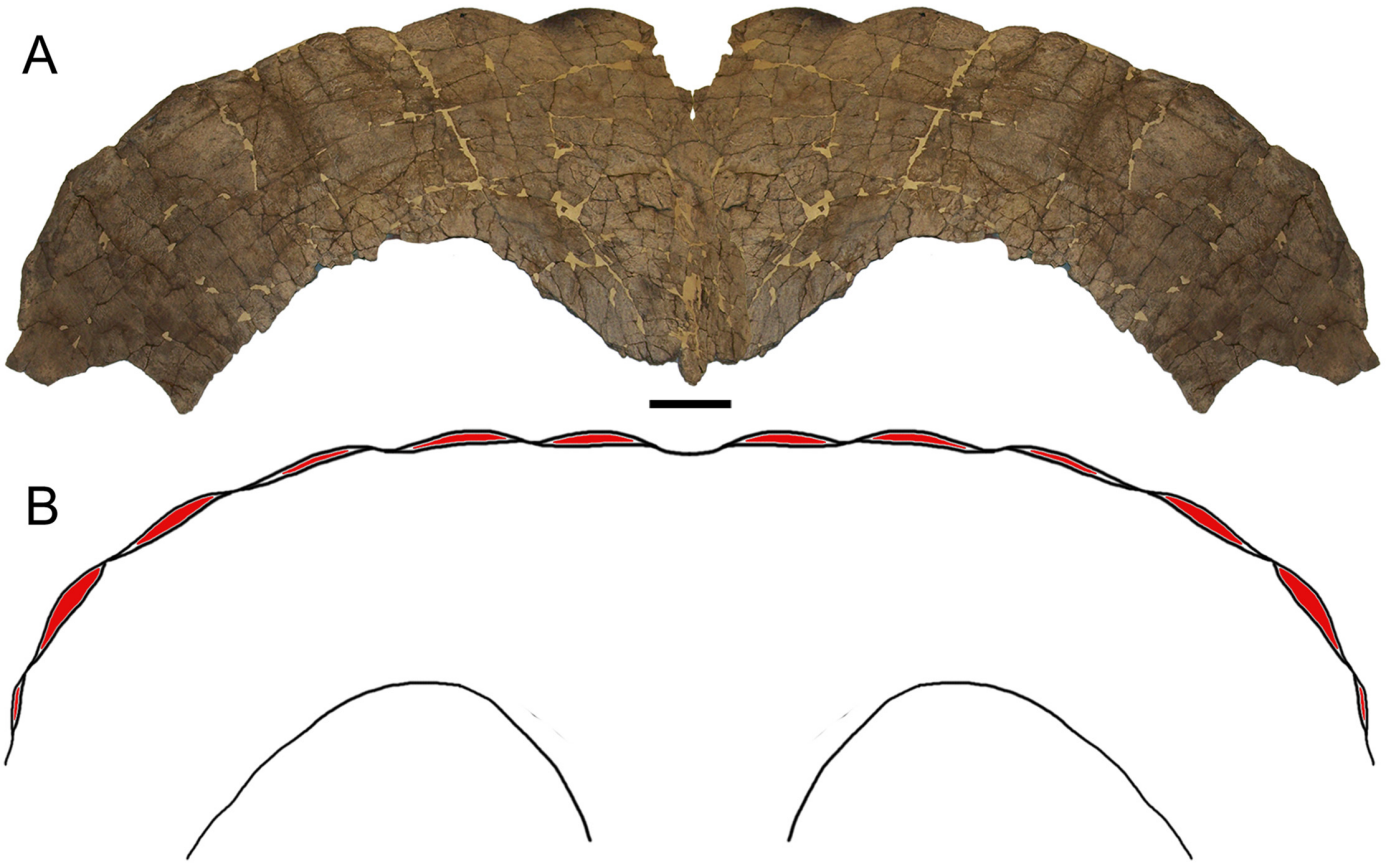
Parietal of *Torosaurus latus*. (A) Image in which ESU 2009–6 has been mirrored horizontally and the two halves aligned along the midline ridge, in dorsal view. (B) Reconstruction of the caudal parietal bar of the individual represented by ESU 2009–6 in dorsal view, with the epiparietals highlighted in red. Scale bar equals 10 cm.

The caudal parietal bar is rostrocaudally broad and gently convex along its caudolateral margin, indicating that the complete parietal would have had a rounded shape (Fig 2), similar to other specimens referred to *Torosaurus latus* (e.g., ANSP 15192, MOR 981, MOR 1122, YPM 1831) [2, 11]. Six epiparietals are present on the caudolateral margin of the parietal, suggesting that the total epiparietal count was 12, as in MOR 1122 [2]. The epiparietals are low, rounded, and slightly rugose (Fig 1B–1D).

## Discussion

### Systematics

*Torosaurus latus* is a derived member of the ceratopsid subclade Chasmosaurinae according to recent phylogenetic analyses that have treated it as a taxon distinct from *Triceratops* [2, 12–15]. Those analyses that have included the other species of *Torosaurus*, *To*. *utahensis* from the North Horn Formation of Utah [16, 17], have recovered different positions relative to *To*. *latus*. Sampson et al. [12], Mallon et al. [13], and Brown and Henderson [15] recovered a monophyletic *Torosaurus* including *To*. *latus* and *To*. *utahensis*, while Longrich [14] found *To*. *utahensis* (as “*Triceratops utahensis*”) to form a clade with *Triceratops horridus* and *Tr*. *prorsus*. The taxonomy and phylogeny of these Maastrichtian chasmosaurines will continue to be refined as additional specimens and new closely related taxa (e.g., *Eotriceratops xerinsularis* [18], *Ojoceratops fowleri* [19], *Regaliceratops peterhewsi* [15]) are discovered.

### Ontogenetic Status

Although histological sampling was not feasible, there are other indications of ontogenetic stage preserved on ESU 2009–6. The dorsal and ventral surfaces of the parietal exhibit rugose, well-vascularized bone texture consistent with a skeletally mature individual [6, 8, 20–24]. Furthermore, the epiparietals are fully fused to the caudolateral margin of the parietal, and the epiparietals themselves are rostrocaudally compressed and contact each other at their bases, features that also indicate maturity [25]. Because of the advanced ontogenetic stage of ESU 2009–6, it cannot significantly contribute to either argument in the ongoing debate over whether *Torosaurus latus* is the old adult form of *Triceratops*. However, it does add another useful data point to the ever-growing chasmosaurine sample from the Hell Creek Formation, which recently was used to explicate a sequence of evolutionary changes in the *Triceratops* lineage from the base to the top of the formation [4]. ESU 2009–6 also provides only the third complete left or right side of the parietal of *Torosaurus latus*, in addition to MOR 981 and MOR 1122 [2].

## Supporting Information

S1 Table Table of Measurements. Select measurements of ESU 2009–6.
(DOC)

## References

[1] Marsh OC. Notice of new vertebrate fossils. American Journal of Science 1891; 42: 265–269.

[2] Farke AA. Cranial osteology and phylogenetic relationships of the chasmosaurine ceratopsid *Torosaurus latus* In: Carpenter K, editor. Horns and Beaks: Ceratopsian and Ornithopod Dinosaurs. Bloomington: Indiana University Press; 2007 pp. 235–257.

[3] Forster CA. Species resolution in *Triceratops*: cladistic and morphometric approaches. Journal of Vertebrate Paleontology 1996; 16: 259–270.

[4] Scannella JB, Fowler DW, Goodwin MB, Horner JR. Evolutionary trends in *Triceratops* from the Hell Creek Formation, Montana. Proceedings of the National Academy of Sciences of the United States of America 2014; 111: 10245–10250. 10.1073/pnas.1313334111 24982159 PMC4104892

[5] Horner JR, Goodwin MB, Myhrvold N. Dinosaur census reveals abundant *Tyrannosaurus* and rare ontogenetic stages in the Upper Cretaceous Hell Creek Formation (Maastrichtian), Montana, USA. PLoS ONE 2011; 6(2): e16574 10.1371/journal.pone.0016574 21347420 PMC3036655

[6] Scannella JB, Horner JR. *Torosaurus* Marsh, 1891, is *Triceratops* Marsh, 1889 (Ceratopsidae: Chasmosaurinae): synonymy through ontogeny. Journal of Vertebrate Paleontology 2010; 30: 1157–1168.

[7] Scannella JB, Horner JR. ‘*Nedoceratops*’: an example of a transitional morphology. PLoS ONE 2011; 6(12): e28705 10.1371/journal.pone.0028705 22194891 PMC3241274

[8] Farke AA. Anatomy and taxonomic status of the chasmosaurine ceratopsid *Nedoceratops hatcheri* from the Upper Cretaceous Lance Formation of Wyoming, U.S.A. PLoS ONE 2011; 6(1): e16196 10.1371/journal.pone.0016196 21283763 PMC3024410

[9] Longrich NR, Field DJ. *Torosaurus* is not *Triceratops*: ontogeny in chasmosaurine ceratopsids as a case study in dinosaur taxonomy. PLoS ONE 2012; 7(2): e32623 10.1371/journal.pone.0032623 22393425 PMC3290593

[10] Maiorino L, Farke AA, Kotsakis T, Piras P. Is *Torosaurus Triceratops*? Geometric morphometric evidence of late Maastrichtian ceratopsid dinosaurs. PLoS ONE 2013; 8(11): e81608 10.1371/journal.pone.0081608 24303058 PMC3841114

[11] Colbert EH, Bump JD. A skull of *Torosaurus* from South Dakota and a revision of the genus. Proceedings of the Academy of Natural Sciences of Philadelphia 1947; 99: 93–106.

[12] Sampson SD, Loewen MA, Farke AA, Roberts EM, Forster CA, Smith JA, et al New horned dinosaurs from Utah provide evidence for intracontinental dinosaur endemism. PLoS ONE 2010; 5(9): e12292 10.1371/journal.pone.0012292 20877459 PMC2929175

[13] Mallon JC, Holmes R, Anderson JS, Farke AA, Evans DC. New information on the rare horned dinosaur *Arrhinoceratops brachyops* (Ornithischia: Ceratopsidae) from the Upper Cretaceous of Alberta, Canada. Canadian Journal of Earth Sciences 2014; 51: 618–634.

[14] Longrich NR. The horned dinosaurs *Pentaceratops* and *Kosmoceratops* from the upper Campanian of Alberta and implications for dinosaur biogeography. Cretaceous Research 2014; 51: 292–308.

[15] Brown CM, Henderson DM. A new horned dinosaur reveals convergent evolution in cranial ornamentation in Ceratopsidae. Current Biology 2015; 25: 1641–1648. 10.1016/j.cub.2015.04.041 26051892

[16] Gilmore CW. Reptilian fauna of the North Horn Formation of central Utah. U.S. Geological Survey Professional Paper 1946; 210-C: 29–52.

[17] Sullivan RM, Boere AC, Lucas SG. Redescription of the ceratopsid dinosaur *Torosaurus utahensis* (Gilmore, 1946) and a revision of the genus. Journal of Paleontology 2005; 79: 564–582.

[18] Wu X, Brinkman DB, Eberth DA, Braman DR. A new ceratopsid dinosaur (Ornithischia) from the uppermost Horseshoe Canyon Formation (upper Maastrichtian), Alberta, Canada. Canadian Journal of Earth Sciences 2007; 44: 1243–1265.

[19] Sullivan RM, Lucas SG. A new chasmosaurine (Ceratopsidae, Dinosauria) from the Upper Cretaceous Ojo Alamo Formation (Naashoibito Member), San Juan Basin, New Mexico In: Ryan MJ, Chinnery-Allgeier BJ, Eberth DA, editors. New Perspectives on Horned Dinosaurs. Bloomington: Indiana University Press; 2010 pp. 169–180.

[20] Sampson SD, Ryan MJ, Tanke DH. Craniofacial ontogeny in centrosaurine dinosaurs (Ornithischia: Ceratopsidae): taxonomic and behavioral implications. Zoological Journal of the Linnean Society 1997; 121: 293–337.

[21] Ryan MJ, Russell AP, Eberth DA, Currie PJ. The taphonomy of a *Centrosaurus* (Ornithischia: Ceratopsidae) bone bed from the Dinosaur Park Formation (upper Campanian), Alberta, Canada, with comments on cranial ontogeny. Palaios 2001; 16: 482–506.

[22] Brown CM, Russell AP, Ryan MJ. Pattern and transition of surficial bone texture of the centrosaurine frill and their ontogenetic and taxonomic implications. Journal of Vertebrate Paleontology 2009; 29: 132–141.

[23] Tumarkin-Deratzian AR. Histological evaluation of ontogenetic bone surface texture changes in the frill of *Centrosaurus apertus* In: Ryan MJ, Chinnery-Allgeier BJ, Eberth DA, editors. New Perspectives on Horned Dinosaurs. Bloomington: Indiana University Press; 2010 pp. 251–263.

[24] Frederickson JA, Tumarkin-Deratzian AR. Craniofacial ontogeny in *Centrosaurus apertus*. PeerJ 2014; 2: e252 10.7717/peerj.252 24688836 PMC3933270

[25] Horner JR, Goodwin MB. Ontogeny of cranial epi-ossifications in *Triceratops*. Journal of Vertebrate Paleontology 2008; 28: 134–144.

