# Supplementary material for: A New Specimen of the Controversial Chasmosaurine Torosaurus latus (Dinosauria: Ceratopsidae) from the Upper Cretaceous Hell Creek Formation of Montana
Source: PLoS One. 2016 Mar 14;11(3):e0151453. doi: 10.1371/journal.pone.0151453 (PMC4790893; doi:10.1371/journal.pone.0151453)
Supplement: S1 Table — Select measurements of ESU 2009–6. (DOC) [file pone.0151453.s001.doc]

**S1, Table of Measurements**

Select measurements of ESU 2009-6.


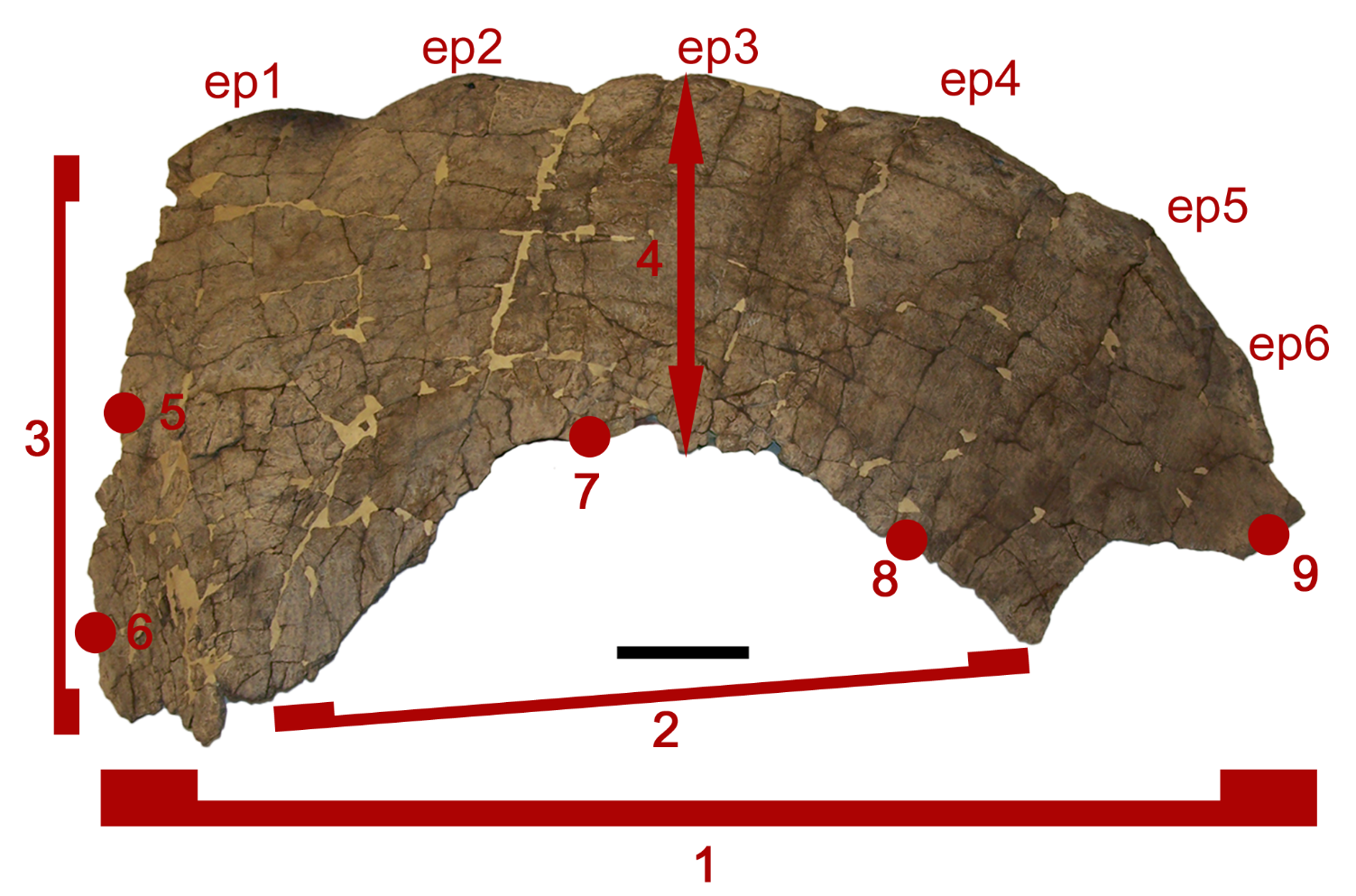


**FIGURE S1. Measurements of ESU 2009-6.** Numbers in figure correspond to measurements in the table. Scale bar equals 10 cm.

| **Measurement** | **Centimeters** |
| --- | --- |
| 1. Preserved mediolateral width | ~95 |
| 2. Preserved width of left parietal fenestra | ~49.5 |
| 3. Preserved rostrocaudal length of midline parietal bar | ~54.5 |
| 4. Preserved rostrocaudal width of caudal parietal bar | ~33 |
| **Parietal thicknesses** |  |
| 5. | 2.7 |
| 6. | 0.4 |
| 7. | 0.5 |
| 8. | 0.4 |
| 9. | 1.2 |
| **Epiparietal dimensions** |  |
| **ep1** |  |
| Mediolateral length | ~17.5 |
| Rostrocaudal length | 3.2 |
| Thickness | 3.0 |
| **ep2** |  |
| Mediolateral length | 16.8 |
| Rostrocaudal length | 2.1 |
| Thickness | 2.6 |
| **ep3** |  |
| Mediolateral length | 20.4 |
| Rostrocaudal length | ~2.7 |
| Thickness | 2.7 |
| **ep4** |  |
| Mediolateral length | 19.4 |
| Rostrocaudal length | 3.5 |
| Thickness | 2.6 |
| **ep5** |  |
| Mediolateral length | ~16.5 |
| Rostrocaudal length | 2.2 |
| Thickness | 2.4 |
| **ep6** |  |
| Mediolateral length | ~11.5 |
| Rostrocaudal length | ~1.8 |
| Thickness | 2.5 |
